# Effect of goji berry incorporation on the texture, physicochemical, and sensory properties of wheat bread

**DOI:** 10.1002/fsn3.4056

**Published:** 2024-02-28

**Authors:** Saba Hashemi, Neda Mollakhalili‐Meybodi, Fateme Akrami Mohajeri, Hossein Fallahzadeh, Elham Khalili Sadrabad

**Affiliations:** ^1^ Research Center for Food Hygiene and Safety, School of Public Health Shahid Sadoughi University of Medical Science Yazd Iran; ^2^ Department of Food Hygiene and Safety, School of Public Health Shahid Sadoughi University of Medical Sciences Yazd Iran; ^3^ Department of Food Science and Technology, School of Public Health Shahid Sadoughi University of Medical Sciences Yazd Iran; ^4^ Infectious Diseases Research Center, Shahid Sadoughi Hospital Shahid Sadoughi University of Medical Sciences Yazd Iran; ^5^ Research Center for Healthcare Data Modeling, Department of Biostatistics and Epidemiology, School of Public Health Shahid Sadoughi University of Medical Sciences Yazd Iran

**Keywords:** enrichment, functional food, goji berry, wheat bread

## Abstract

The regular intake of *Lycium barbarum* (goji berry) is supposed to play an important role in the promotion of human health. Regarding, its incorporation into staple foods, including bread, seems to be effective. However, it requires the evaluation of dough behavior and final product quality. This study investigated the effect of goji berry incorporation at levels of 10, 15, 20, 25, and 30% ww^−1^ on the textural, physicochemical, and sensory properties of wheat bread. Results indicated a significant enhancement of water absorption and gelatinization temperature in composite flour via the inclusion of goji berry powder (*p* < 0.05). Using goji berry powder up to 20% ww^−1^ has shown to obtain the structure able to restore gases through the baking process and provide enhancement in a specific volume at about 10%. Alongside, the hardness of composite bread decreased, and the optimal hardness was observed at formulations containing 20% w/w goji berry powder with a value equal to 1199.95 ± 0.05 g, which is supposed to be induced by the higher specific volume and lower moisture content of bread samples. Moreover, color and sensory perception have been found to be significantly changed by goji berry substitution. Goji berry substitution up to 20% ww^−1^ is found to be preferred by the consumer, and a drop in overall acceptability was observed at its higher inclusion. The technological characteristic changes induced by goji berry incorporation are induced by its gluten dilution impact. However, the gel‐like structure formed by the high fiber content of goji berries compensates for this adverse impact up to 20% w/w substitution level.

## Graphical abstract

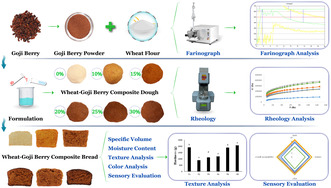

## 1 INTRODUCTION

The fruits of *Lycium barbarum*, popularly known as goji berry or wolfberry, are mostly grown in China, Tibet, and other Asian nations (Koçyiğit & Şanlier, 2017). It is ellipsoid‐shaped, bright red in color, and approximately 1–2 cm long, with a sweet and tangy flavor. The berries are commonly used in a variety of products like soup, juice, pastries, and tea in dried forms that resemble raisins (Koçyiğit & Şanlier, 2017). To improve the nutritional content of foods, exotic and rare fruits (and the products generated from them) such as goji berries are being employed more frequently in food formulation. The goji berry is renowned for its numerous nutritional values and high carbohydrate, protein, fat, dietary fiber, and micronutrient content (Skenderidis et al., 2019). Berries also offer positive health impacts, including immunomodulation and anticancer activity, body weight reduction (Wenli et al., 2019), blood sugar/pressure regulation, and protective effects on retina cells. Additionally, because of its high non‐digestible polysaccharide content, it is helpful for people with diabetes mellitus (Endes et al., 2015).

Functional foods refer to foods in which their harmful compounds are removed or useful compounds are added. Therefore, functional foods have higher beneficial nutritional properties and positive effects on health compared to their natural state through regular and proper consumption (Kowalczewski et al., 2019). The addition of goji berry by‐products (10, 20, 30, and 40 g/100 g) in muffins and cookies increased the dietary fibers, protein, and free phenolics. The best sensory characteristics were reported at 30 and 20 g replacement of goji berry by‐product (Bora et al., 2019). Enrichment of bread with 50% and 70% goji puree showed higher total phenolic content and antioxidant activity, especially at the 50% level (Loizzo et al., 2021). Considering the benefits mentioned for goji berries, their addition as an ingredient in the production of functional foods could amplify its beneficial characteristics. By increasing human knowledge about the direct impacts of diet on health and their growing desire to consume functional foods, fortification of staple food formulations with suitable ingredients is considered as the most convenient and straightforward solution (Balabanova et al., 2018). Wheat bread is a staple foodstuff produced and consumed in most countries around the world, with specific technological characteristics induced by a unique three‐dimensional gluten network (Mohammadi et al., 2021). Any fortification of wheat bread could potentially influence its technological characteristics, which are induced by gluten dilution impacts (Meybodi et al., 2019). Consequently, the potential of wheat bread to deliver functional ingredients like goji berries needs to be monitored from a technological characteristics perspective. Therefore, in the current study, the technological characteristics of wheat bread in the presence of goji berry flour were investigated by measuring the farinograph and rheological properties of the dough and physicochemical characteristics, texture profile, crumb/crust color, and sensory evaluation of the bread.

## 2 MATERIALS AND METHODS

### 2.1 Sample procurement

For this experiment, ripened goji berry fruits (*Lycium barbarum*) cultivated in Iran were purchased in the form of dried fruit from an Iranian berry company (Mazandaran, Iran). Then the dried goji berry was grounded using an electric blender. The sieving process was done by passing them through two sieves with 50 and 40 mesh sizes. Soft wheat flour was procured at Roshan Company (Yazd, Iran), containing 13.95% ww^−1^ moisture, 0.23% ww^−1^ ash, and 10.93% ww^−1^ protein. Other materials, such as fresh yeast, salt, sugar, and oil, were purchased from the local market of Yazd, Iran.

### 2.2 Method

The potential of wheat bread to deliver goji berries was investigated regarding technological characteristics (farinograph and rheological properties of dough, texture profile analysis, crumb/ crust color, and sensory evaluation). Goji berry bread samples were prepared according to the procedure described by Gamel et al. (2015). The composite flour was prepared by replacing part of wheat flour with grounded goji berry (the moisture, ash, fat, protein, fiber, and carbohydrate were 6.9, 4.11, 3.75, 13.21, 24.81, and 402.31 g/100 g DW, respectively) at 6 levels (0, 10, 15, 20, 25, and 30% ww^−1^) as described in Table 1. The quantity of water was determined using the Farinograph (Brabender, Germany), and breads were fermented with *Saccharomyces cerevisiae* yeast as a starter.

**TABLE 1:** The proportion of wheat flour and goji berry powder in the formulation of composite flour.

Number	Trial	Proportion	
		Wheat flour (g/100 g)	Goji berry powder (g/100 g)
1	F_1_	100	0
2	F_2_	90	10
3	F_3_	85	15
4	F_4_	80	20
5	F_5_	75	25
6	F_6_	70	30

### 2.3 Farinograph test

The mixing properties of composite flour were determined by the Brabender farinograph (Germany) using the method described in AACC Method 5421 (Kou et al., 2019). The determined parameters from the curves plotted by the device were: water absorption (WA), dough development time (DDT), stability time (ST), dough degree of softening (DDS), consistency, and farinograph quality number (FQN). Values were expressed as the mean ± standard deviation.

### 2.4 Bread preparation

**FIGURE 1:**
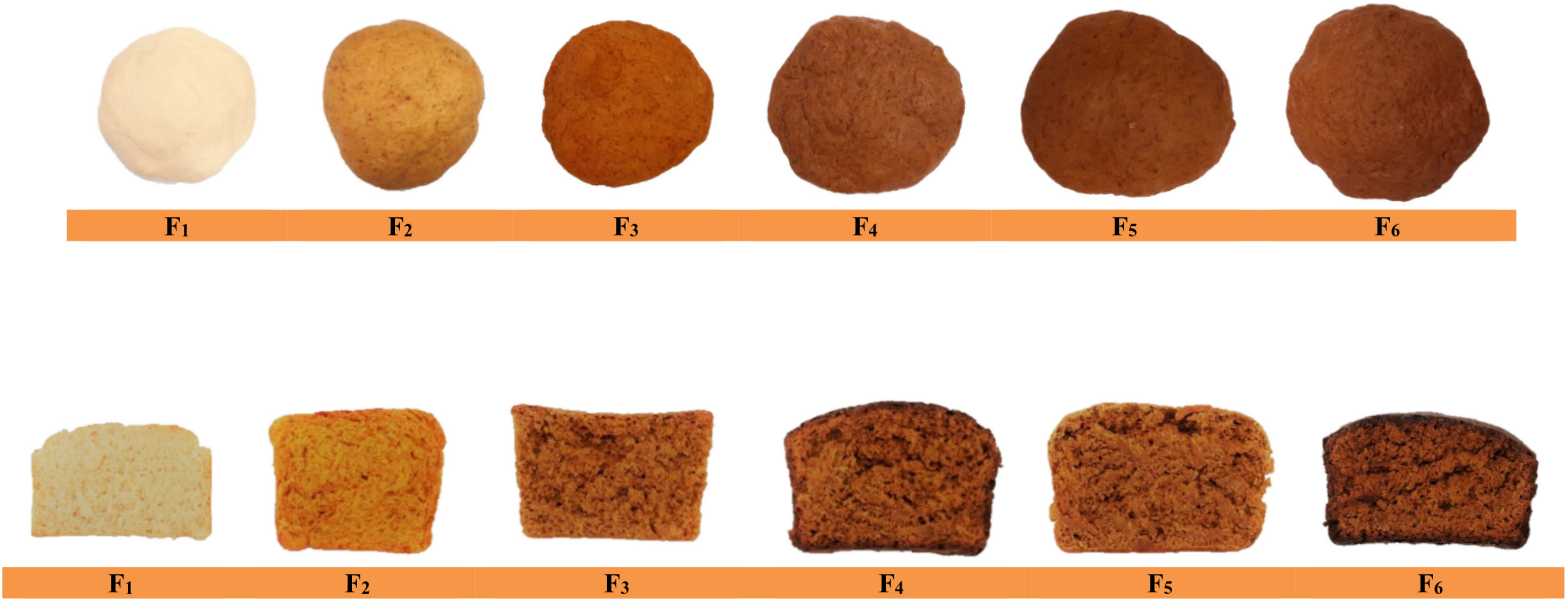
The appearance of the dough samples – cross section of bread samples prepared in the present study.

The dough preparation was done using soft wheat composite flour, in which goji berries were incorporated at 6 levels (0, 10, 15, 20, 25, and 30% ww^−1^). All ingredients were added on a flour‐dry basis. In this regard, 2.2% ww^−1^ yeast, 0.5% ww^−1^ sugar, 1% ww^−1^ salt, 3% ww^−1^ canola oil, and water (based on farinograph data) were mixed. Then, the mixture was kept inside the fermentation cabinet set at 29 ± 0.5°C for 4 h. A piece of sample (30 g) was kept for rheological parameter determination, and the other was used for baking. Afterward, the kneaded dough was placed in baking pans and baked in a convection oven (Model PFB‐2, Duke Manufacturing Company, St Louis, MO, USA) at 220°C until easily separated from the pan edges. The schematic diagram of wheat–goji berry composite bread preparation steps is declared in Figure 1.

### 2.5 Rheological characteristic of wheat–goji berry composite dough

The rheological characteristics of wheat–goji berry composite dough were determined by the method described by Codină et al. (2018). For this purpose, a Physica MCR 301 rotary rheometer (Austria, Anton Parr) equipped with a circular water temperature regulation system with a sensitivity of ±0.01 with a parallel plate measuring system and a gap width of 2 mm was used. Dough samples were prepared according to the optimum water absorption capacity determined by Farinograph. Dough samples rested for 30 min to allow relaxation and temperature stabilization before being placed between plates. To determine the linear viscoelastic region, frequency sweep tests were measured from 1 to 20 Hz at 20°C for all wheat–goji berry composite dough samples. For the temperature sweep test, samples were heated from 20 to 85°C at a heating rate of 4.74°C per min and a frequency of 1 Hz. Also, rheological parameters including storage modulus (*G*′), loss modulus (*G*″), and loss tangent (tan δ) were registered as follows:

$$\tan\delta =\frac{G^{\prime }}{G^{\prime \prime }}.$$

### 2.6 Physicochemical characteristics of wheat–goji berry composite bread

#### 2.6.1 Specific volume

The bread was determined for volume 1 h after baking using the rapeseed displacement method, following the guidelines provided by the AACC Method (McCleary et al., 2013). Then the specific volume was calculated in cm^3^/g by using the following formula:

$$\text{Specific volume}=\frac{\text{Volume of bread}}{\text{Weight of bread}}.$$

#### 2.6.2 Moisture content

Based on the procedure described by AACC44‐16, the moisture content of the bread samples was quantified using the oven drying method, in which 5 g of the sample were placed in a metal dish that was closed and weighed. The dish was placed in an oven at 100°C for 5 h, and after cooling down, it was weighed once again until a constant weight was obtained. The moisture was calculated by subtracting the final weight from the initial one (American Association of Cereal Chemists. Approved Methods Committee, 2000).

#### 2.6.3 Ash content

Ash content was determined according to AACCI method 08–01.01, in which 10 g of the sample were incinerated in a muffle at 550°C until the obtained ash became white or gray in color. The mineral content was expressed as g/100 g of fresh weight (American Association of Cereal Chemists. Approved Methods Committee, 2000).

### 2.7 Texture profile analysis

The textural characteristics of all wheat–goji berry composite bread were measured on the day of baking using a texture analyzer (TA20. KOOPA) (AAC, 2000). Textural characteristics, including hardness, cohesiveness, springiness, and chewiness, of bread samples were evaluated. Bread loaves were sliced from the middle into pieces of 20 × 20 × 25 mm. The bread samples were compressed to up to 50% of their original height through a 43‐mm cylindrical probe using a 5‐kg loading cell at a speed of 1 mm/s. The parameters were calculated using Texture Expert software for Windows (Guadarrama‐Lezama et al., 2016).

### 2.8 Color analysis

The crust and crumb color of bread samples were evaluated using a spectrophotometer UV–Visible (Varian, Australia). The spectral attributes were based on the CIE system, lightness (L*), greenness/redness (a*), and blueness/yellowness (b*). The CIE whiteness index of each bread sample was calculated by the following equation (Zhu et al., 2016):

$$\text{Whiteness index}=100-\sqrt{{\left(100-L*\right)}^{2}+{\left(a*\right)}^{2}+{\left(b*\right)}^{2}}.$$

### 2.9 Preliminarily sensory evaluation

The sensory characteristics of the loaves of bread were evaluated by 30 consumers aged between 20 and 50 years. Participants were recruited from the staff and students at Shahid Sadoughi University of Medical Sciences, Yazd, Iran. Samples were given to the panelists (who were insensitive to gluten/wheat products) randomly. Six samples of bread (0, 10, 15, 20, 25, and 30% ww^−1^) and water (25°C) were provided to the panelists, and they were also requested to rinse their mouths with plain water between each sample tasting. Different sensory attributes of the product, namely, texture, flavor/taste, color, and overall acceptability, were evaluated by the panelists. The sensory scores of bread were recorded using the 9‐point hedonic scale (1 = dislike extremely; 2 = dislike very much; 3 = dislike moderately; 4 = dislike slightly; 5 = neither like nor dislike; 6 = like slightly; 7 = like moderately; 8 = like very much; 9 = like extremely) (Bora et al., 2019).

### 2.10 Statistical analysis

In order to obtain the highest accuracy in the results, each test was performed in triplicate. Statistical analysis was carried out by one‐way ANOVA using SPSS V.25 software. Significant differences between mean values were determined by Tukey's test. Furthermore, non‐parametric tests were used for sensory evaluation (Kruskal–Wallis test). *p* values <0.05 were considered significant.

## 3 RESULT AND DISCUSSION

### 3.1 Farinograph test result

The unique properties of wheat flour in the formation of a viscoelastic network were influenced by the addition of other ingredients. Table 2 shows the farinograph results of the wheat dough (control) and wheat–goji berry composite dough. Results showed that goji berry inclusion changed the mixing behavior of wheat dough on the basis of its level. Considering the water absorption index (WAI), a significant (*p* < 0.05) increase has been observed by goji berry inclusion from 66.41 ± 0.01 i to 68.44 ± 0.02 in the control and F_2_ samples, respectively. However, an insignificant descending WAI was observed by increasing the quantity of goji berry inclusion between F_4_, F_5_, and control samples. Also, the lowest WAI (*p* < 0.05) was found at the F_6_ formulation containing the highest goji berry powder content. It is shown that the amount of protein and fiber in fruit could change the WAI of flour by influencing the gelatinization process (Mohammadi et al., 2021). Gluten protein has a higher water absorption rate compared to fibers (Struck et al., 2018). However, the compliance role of fiber with protein in water absorption postpones the decrease in WAI in formulations containing goji berries up to 25% ww^−1^, which is obvious at F_6_, verifying the gluten dilution impacts (Shiri et al., 2021).

**TABLE 2:** Farinograph characteristics of wheat–goji berry composite flour.

Trial	Parameters				
	WA (g/100 g)	DDT (min)	ST (min)	DDS (FU)	FQN (−)
F_1_	66.41 ± 0.01^c^	4.91 ± 0.01^c^	18.94 ± 0.04^a^	123.01 ± 0.01^f^	68.01 ± 0.01^b^
F_2_	68.44 ± 0.02^a^	3.44 ± 0.02^d^	7.45 ± 0.02^d^	280.95 ± 0.01^c^	42.95 ± 0.02^c^
F_3_	67.45 ± 0.02^b^	1.65 ± 0.01^e^	8.55 ± 0.02^c^	235.97 ± 0.02^d^	17.97 ± 0.02^d^
F_4_	66.97 ± 0.01^c^	6.85 ± 0.01^c^	14.21 ± 0.07^b^	192.95 ± 0.01^e^	77.95 ± 0.02^b^
F_5_	67.02 ± 0.01^c^	9.93 ± 0.01^a^	19.15 ± 0.01^a^	624.96 ± 0.02^a^	107.94 ± 0.01^a^
F_6_	65.25 ± 0.02^d^	8.44 ± 0.02^b^	19.25 ± 0.02^a^	550.94 ± 0.01^b^	102.97 ± 0.02^a^

Abbreviations: DDS, dough degree of softening; DDT, dough development time; FQN, farinograph quality number; ST, stability time; WA, water absorption.

*Note*: Data are reported as average ± standard deviation. Values with different lowercase letters, according to Tukey's test, are significantly different in each column (*p* < 0.05).

Dough development time (DDT), as an indicator of the time required to form dough with suitable consistency, is prominently influenced by protein and fiber content in formulation (Liu et al., 2022). As depicted in Table 2, the DDT amount change by goji berry powder inclusion was significant (*p* < 0.05). Despite the decrease observed by goji berry addition at 15% ww^−1^, it has been increased again at the higher incorporation level, where the highest DDT content is observed in the F_5_ sample with a value equal to 9.93 ± 0.01 min. It seems that increasing the amount of fruit‐based dietary fiber in dough samples reduces the hydration of wheat proteins and increases DDT (Struck et al., 2018). As DDT is an indicator of the time required for dough development (Tamba‐Berehoiu et al., 2017), it is preferred to be similar to control through fortified formula optimization (Jagelaviciute & Cizeikiene, 2021). In this regard, the closest content in composite dough is found at F_4_, with an insignificant difference from the control (*p* ≥ 0.05), verifying the compensation of gluten dilution impacts with goji berry dietary fiber (Liu et al., 2022). The optimized stability time (ST), which displays dough stability and strength, is also found in F_4_ containing goji berries at 20% ww^−1^ (Kohajdová et al., 2014).

Respecting dough degree of softening (DDS), an increase of about 300% has been found at 30% ww^−1^ goji berry inclusion in composite dough. Although no particular trend has been found in this parameter, the lowest DDS in composite dough was found in F_4_. The lowest DDS along with optimized ST at composite dough confirm the compensation role of dietary fiber on the gluten dilution role of gluten at composite dough. In general, a high degree of softening indicates a weak dough, in which most of the dough stabilizing bonds have been broken and caused water to leave the system (Iuga & Mironeasa, 2020).

By using goji berry powder in composite dough, a reduction of about 70% is found at FQN of the F_3_ sample containing 15% ww^−1^ goji berry, which is induced by a decrease in ST and an increase in DDS of the sample. As FQN is an indicator of the quality of flour, it needs to be close to the control sample at fortified formulations, which is an indicator of the desirability of the formulation (Valková et al., 2020). Among composite doughs, F_4_ sample was the closest sample in terms of FQN. Similar results were reported by Kim et al. (2019) by adding grape seed powder to wheat flour.

### 3.2 Rheological characteristics

**FIGURE 2:**
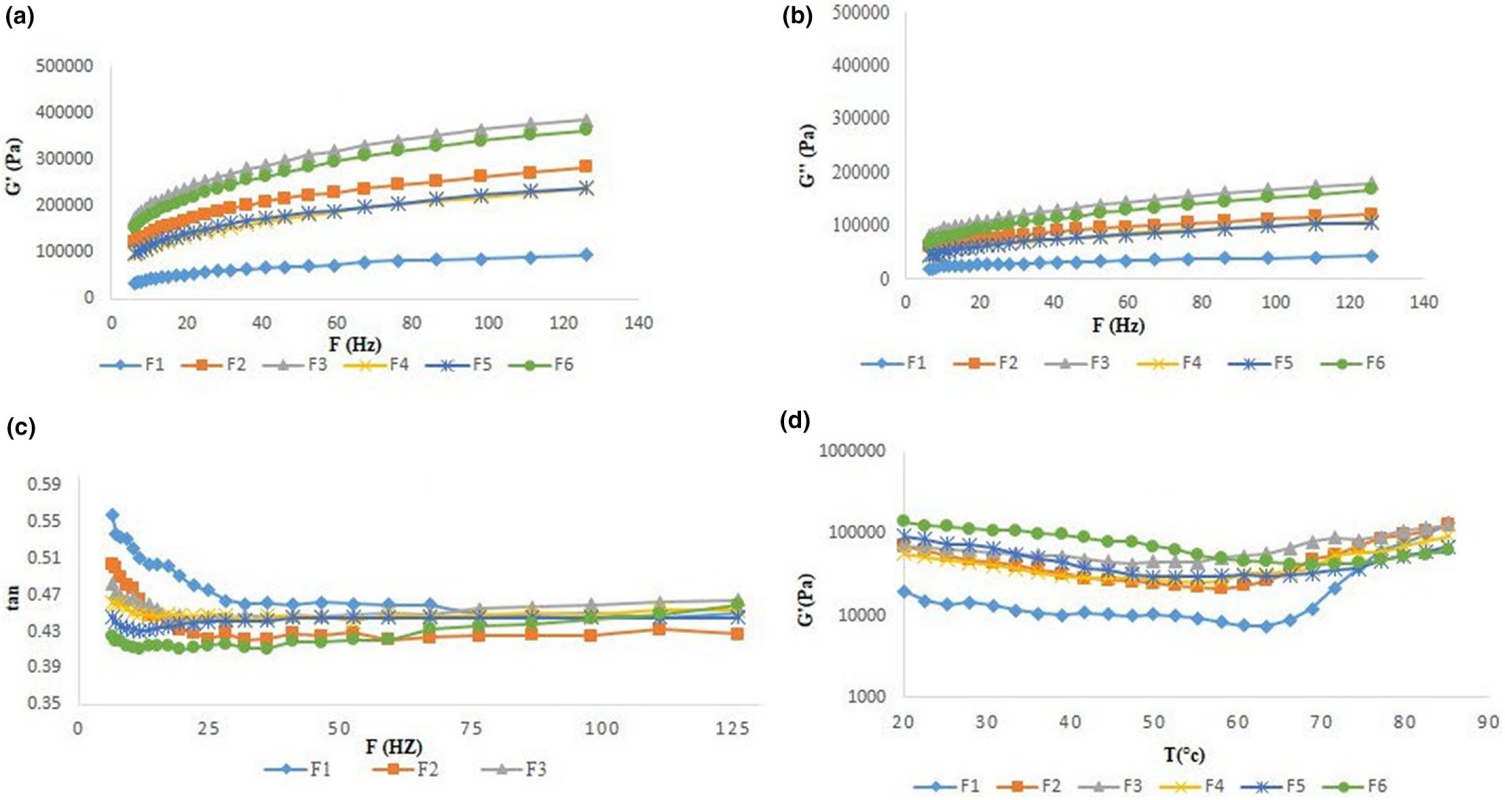
(a) Storage (G′) and (b) loss modulus (G″) variations with frequency for goji berry containing samples. (c) Loss tangent (tan δ) variation with frequency for goji berry containing samples. (d) Effect of goji berry replacement on the changes of storage modulus (G′) vs. temperature.

Frequency sweep and temperature sweep tests provide information about the effect of changes in formulation on the behavior of the dough during the process (Korus et al., 2017). Tests were performed at a linear viscoelastic region, where the viscoelastic behavior of the materials is strain‐independent, in order to determine the maximum gelatinization temperature (Iuga et al. 2020). The viscoelastic characteristics of dough are monitored by two viscose and elastic moduli, which are assessed by complex modulus and damping factor parameters (Witczak et al., 2017). Regarding the data obtained by the frequency sweep test, the storage modulus was greater than the loss modulus (G′ > G″) at all complex doughs. As depicted in Figure 2, using goji berry powder in composite dough leads to an increase in both elastic and viscose moduli (with an enhanced increase in elastic modulus as demonstrated by a decreased damping factor), depending on the incorporation level. Therefore, using goji berries in composite dough formulations provides a gel‐like structure with more elastic behavior compared to viscose characteristics (Valková et al., 2020). However, this trend is found to be dependent on goji berry level, with an increasing trend up to F_3_ and again decreasing. In other words, the impacts of gluten dilution will be more dominant in formulations containing more than 15% ww^−1^ goji berries. Regarding complex modulus, among complex doughs, the behavior found in F_4_ is more similar to that of the control sample. High‐quality dough needs to provide optimal complex modulus, as those with high complex modulus prevent gas bubble expansion and are characterized by excessive stiffness, and those with low complex modulus are poor enough to be unable to maintain gas bubbles (Shiri et al., 2021).

Temperature sweep test indicate information about the changes of the complex modulus with temperature. These changes are owing to the effects of temperature on the moisture absorption rate (Figure 2). Also, changes of elastic modulus is related to starch, dietary fiber, protein content, and its ratio (Struck et al., 2018). It has been found that goji berry addition changes the moisture absorption rate and initial viscosity. The gelatinization temperature and the manner of elastic modulus would change over time. The viscosity at 20°C showed that the addition of goji berries had a high effect on the viscosity, and with the addition of goji berries, viscosity significantly increased. But with increasing temperature to 70°C, the viscosity of all systems reduced. After that, with an increase in temperature, viscosity increased due to the absorption of water. The absorption rate of water decreased with an increase in the content of goji berries owing to the reduction in the granola content of the systems. As demonstrated, the gelatinization temperature has been increased by the addition of goji berry in a dose‐dependent manner, increasing from 66°C in the control sample to about 90°C in the formulation containing 30% ww^−1^ goji berry. As gelatinization occurs by water absorption of starch molecules, it seems that in the presence of goji berry fiber and its competition with starch molecules, the water absorption of starch and consequently its gelatinization will delay (Xu et al., 2021).

### 3.3 Physicochemical characteristics

Moisture plays an important role in the formation of dough and the quality of bread texture. It depends on the components and structure of the formulation and is influenced by the absorption water layer, bound water layer, and free water layer (Liu et al., 2022). According to the results presented in Table 3, the inclusion of goji berry powder significantly reduced the moisture content of composite bread samples compared to the control ones (*p* < 0.05), which is promoted by increasing the goji berry powder incorporation level. Considering both the water absorption and moisture content, they have changed in a similar way. In other words, the lowest and highest moisture content have been observed in samples with the lowest and highest water absorption content, respectively (F_6_ and F_2_). Moisture reduction of composite dough at high levels of goji berry inclusion is attributed to a reduction in the dehydration of dough structure as a result of the weakening of the gluten network (Ahmed et al., 2013). Also, the lowest moisture content was observed in the sample containing 30% ww^−1^ (F_6_) with 31.69 ± 0.01% ww^−1^. It seems that the decrease in moisture content is due to the amount of fiber in the fruit composition, which competes with starch molecules and reduces its dehydration. As can be seen from the results of the temperature sweep test, in the higher concentrations of goji berries, the gelatinization of the starch delays, and the gelatinization temperature eventually increases. These changes prevent the formation of a regular gel‐like structure and cause more water migration and less moisture content (Ragaee et al., 2011).

**TABLE 3:** Physicochemical characteristics of wheat–goji composite bread.

Trial	Parameters		
	Moisture (g/100 g)	Specific volume (cm^3^/g)	Ash (cm^3^/g)
F_1_	39.09 ± 0.02^a^	2.18 ± 0.01^b^	1.11 ± 0.01^e^
F_2_	36.20 ± 0.02^b^	2.39 ± 0.01^a^	1.37 ± 0.01^d^
F_3_	35.12 ± 0.02^c^	2.40 ± 0.01^a^	1.51 ± 0.01^c^
F_4_	33.20 ± 0.02^d^	2.40 ± 0.01^a^	1.72 ± 0.01^b^
F_5_	31.78 ± 0.02^e^	2.05 ± 0.03^d^	1.74 ± 0.01^b^
F_6_	31.69 ± 0.01^e^	2.08 ± 0.02^c^	1.92 ± 0.02^a^

*Note*: Data are reported as average ± standard deviation. Values with different lowercase letters, according to Tukey's test, are significantly different in each column (*p* < 0.05).

The specific volume is an index of the dough's ability to retain the carbon dioxide produced during the fermentation process and its ability to expand it during the baking process (Kou et al., 2019). The specific volume depends on the quality and quantity of wheat flour constituents (such as protein, starch, dietary fiber, etc. content and ratio) and composite dough formulation (Bora et al., 2019). Results indicated that despite a significant increase in the specific volume of bread samples by goji berry powder incorporation up to 20% ww^−1^ (sample F_4_) (*p* < 0.05), it decreased at higher incorporation levels (samples F_5_ and F_6_) (*p* < 0.05). Therefore, it was hypothesized that inhibitory impacts of dietary fiber in formulations containing high goji berry levels decrease the specific volume (Pathak et al., 2016). With an increase in goji berry concentration, the moisture absorption and moisture content of the sample significantly decreased. The highest specific volume of bread samples was observed in the mixture with 20% ww^−1^ of goji berry powder, which increased by about 10% compared to the control sample. Also, the water absorption was reduced in this sample, which led to moisture migration. The effect of adding goji berry powder on the specific volume of bread samples also depends on the amount of gluten protein dilution. This could be clarified by the fact that high substitution levels of bread samples with goji berry powder cause gluten dilution, affect optimal gluten matrix formation, and verify the formation of compact structures (Feili et al., 2016).

The amount of ash content is an estimate of the mineral content in the food (Martínez & Gómez, 2017). Results indicated that goji berry addition enhanced the ash content according to its incorporation level, which is in accordance with Bora et al.'s (2019) results.

### 3.4 Textural characteristics

**FIGURE 3:**
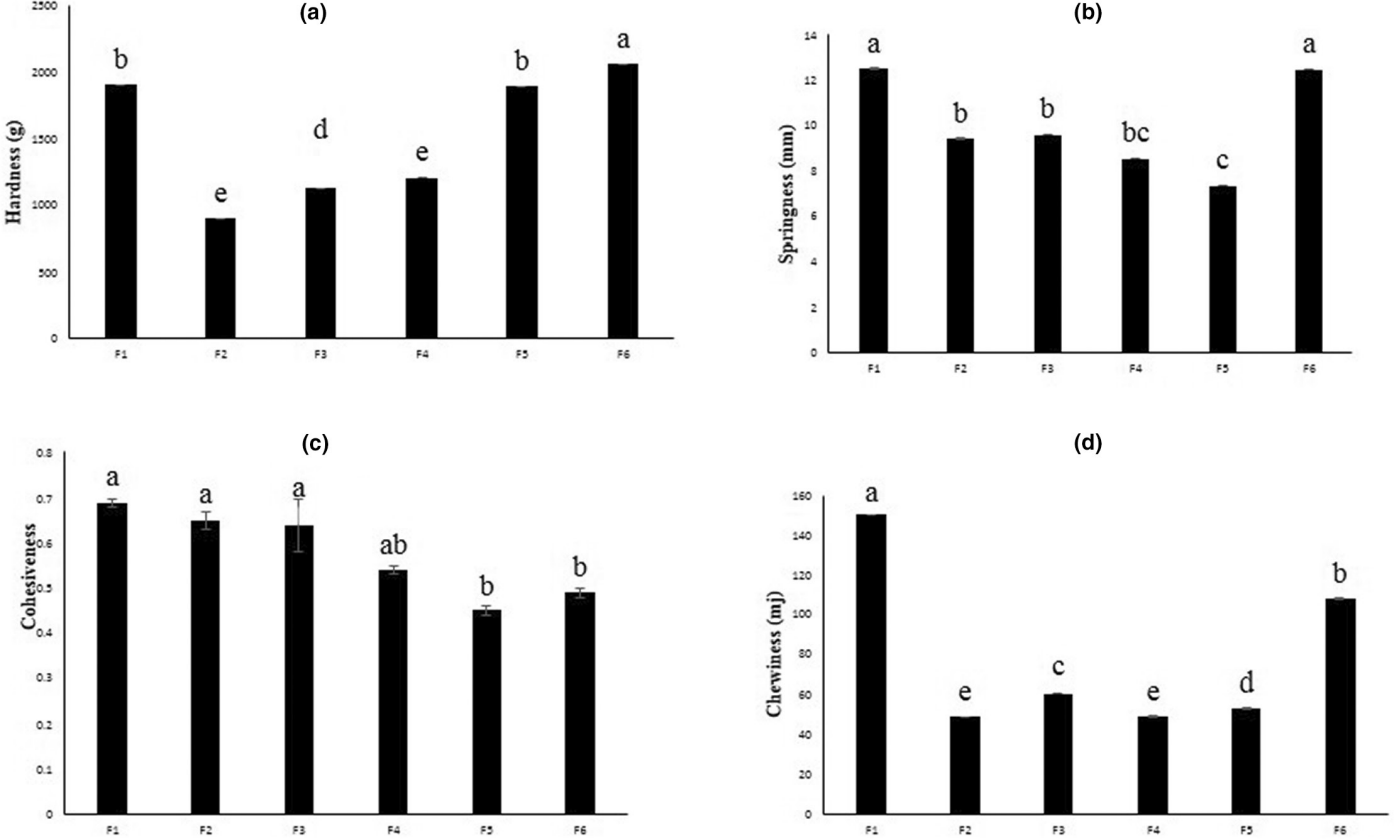
Hardness (a), springiness (b), cohesiveness (c), and chewiness (d) of wheat breads containing goji berry powder. Data are reported as average ± standard deviation.

The textural properties of composite breads in the presence of goji berry powder are shown in Figure 3. According to the results, hardness of the samples ranged from 892.23 ± 0.37 to 2068.17 ± 0.03 g. Generally, the hardness of bread indicates its quality and depends on specific volume, dough hydration, and formulation components such as protein and fiber (Shittu et al., 2014). It is clear that hardened bread can reduce consumer acceptance (Liu et al., 2022). Incorporation of goji berry powder at levels up to 20% ww^−1^ (F_4_) significantly decreased the hardness of composite breads (*p* < 0.05). Decreased hardness, along with a higher specific volume and lower moisture content, of bread samples seems to be induced by a higher water migration ratio, starch gelatinization, the presence of fiber, and an optimal fiber–protein ratio affect that water migration (Feili et al., 2013). Based on the result, sample F_4_ is considered optimal in terms of hardness, which is the same as the control sample.

Springiness (known as elasticity) is an indicator of the quality and freshness of bread and is expressed as the degree to which deformed breads return to their normal state after being pressed (Lauková et al., 2017). Springiness was significantly decreased by adding goji berry powder (*p* < 0.05). The highest elasticity was reported in the control sample, which showed an insignificant difference with the sample containing 30% ww^−1^ of goji berry powder (*p* ≥ 0.05). The high springiness in the control sample could be attributed to the dilution of the gluten structure in the substituted breads. The lower amount of gluten causes a lower ability to hold the gases, which causes a reduction in the springiness of composite breads (Amin et al., 2019). The interaction between gelatinized starch and gluten causes the dough to be more elastic and can be effective in maintaining the spongy structure of bread after the baking process (Mildner‐Szkudlarz et al., 2016). Results also indicated that at higher levels of goji berry powder, an increase in the amount of fiber and a decrease in the amount of protein in the final formulation will increase the gelatinization time and reduce the ability to hold gases. These changes would ultimately lead to a decrease in the elasticity of bread.

Cohesiveness as an indicator of bread quality (Kohajdová et al., 2014) has a range of 0.45 ± 0.01–0.69 ± 0.01. High cohesiveness and elasticity for wheat bread is a desirable feature because it leads to less distribution of bread during chewing. In fact, low cohesiveness in bread is susceptible to rupture (Encina‐Zelada et al., 2018). Results showed that, using goji berry powder up to 20% ww^−1^ (F_4_ sample) has insignificant impacts on the cohesiveness of samples (*p* ≥ 0.05). However, at a higher substitution level of goji berry powder, cohesiveness decreased, and an insignificant difference was also observed between samples F_5_ and F_6_ (*p* ≥ 0.05). The decrease in cohesiveness seems to be induced by gluten dilution impacts at higher incorporation levels of goji berries. Higher substitution levels of composite breads have a low ability to resist the bite force between the teeth before the bread structure breaks and are more likely to disintegrate during masticating. Chewiness shows the energy required to chew a solid food until it can be swallowed (Kowalczewski et al., 2019) and considers the parameters of hardness, cohesiveness, and elasticity simultaneously. The chewiness of breads has increased with an increase in the amount of goji berry powder, in a trend similar to hardness. It was shown that among breads enriched with goji berry powder, the highest chewiness was found in F_6_ samples. The correlation between chewiness and hardness was also found by Feili et al. (2016). Overall, the addition of goji berry powder to the bread recipe, due to gluten dilution and dough's low ability to hold gas, led to the weakening of the gluten structure; as a result, the softness and springiness of composite bread were reduced and chewiness increased (Hameed et al., 2021). A study by Feili et al. (2016) showed a similar trend for breads containing fiber, in which the addition of fiber to the formulation increased the chewiness of the bread.

### 3.5 Color analysis

The effects of goji berry powder addition on composite bread color are presented in Table 4. Bread color is an important quality parameter of bakery products, which, along with texture and taste, could have a significant impact on their acceptability. The color of the golden crust and the color of the white bread crumb are the factors affecting consumer acceptance (Guijarro‐fuertes et al., 2019). During the processing of goji berry‐wheat composite bread, the reddish‐orange color of the fruit provides a red range of color to the dough and bread. Incorporation of goji berry powder in the formulation of wheat bread led to a decrease in L* (lightness), a* (greenness/redness), and b* (blueness/yellowness) of the bread crust. The control sample presented a lighter crust compared to goji berry‐enriched bread, and it became darker by increasing the substitution level of goji berry powder. Among the samples that contained goji berry powder, those with 10% ww^−1^ goji berry powder (F_2_) with values equal to 83.70 ± 0.30, 12.34 ± 0.01, and 24.13 ± 0.01 had the highest L*, a*, and b*, respectively. With an increase in goji berry level, all crust color parameters have decreased, with insignificant differences between samples F_3_, F_4_, F_5_, and F_6_ (*p* ≥ 0.05). As can be seen, the crust color is influenced by the Maillard reaction (Mohammadi et al., 2021). According to the results, the incorporation of goji berry powder reduced the L* and a* together. However, L* reduction can be attributed to the accelerated Maillard reaction induced by enhanced substrate availability and the reddish color of goji berry powder (Eliášová et al., 2020). In fact, no certain reason existed for a* reduction despite the prevalence of goji berry color. Similar results were obtained by Guijarro‐fuertes et al. (2019), in which the use of Andean blueberries in wheat bread reduced the lightness (L*), redness (a*), and yellowness (b*) of the bread crust. Also, the WI crust changes showed that the use of goji berry powder increased the whiteness of bread crust and between samples F_3_, F_4_, F_5_, and F_6_, which was insignificant (*p* ≥ 0.05).

**TABLE 4:** Crust and crumb color analysis of wheat–goji berry composite bread.

Trial	Crust				Crumb			
	L*	a*	b*	WI	L*	a*	b*	WI
F_1_	86.71 ± 0.08^a^	12.15 ± 0.62^a^	27.37 ± 0.04^a^	67.23 ± 0.18^c^	90.75 ± 0.02^a^	6.28 ± 0.02^d^	27.03 ± 0.17^a^	70.74 ± 0.12^a^
F_2_	83.70 ± 0.30^b^	12.34 ± 0.01^a^	24.13 ± 0.04^b^	68.37 ± 0.09^b^	85.42 ± 0.13^b^	12.74 ± 0.06^b^	27.62 ± 0.30^a^	66.26 ± 0.17^c^
F_3_	80.90 ± 0.15^c^	9.75 ± 0.03^b^	21.21 ± 0.31^c^	69.83 ± 0.10^a^	84.67 ± 0.33^b^	12.97 ± 0.23^b^	26.54 ± 0.20^a^	66.71 ± 0.07^c^
F_4_	81.92 ± 0.08^c^	10.55 ± 0.02^b^	21.59 ± 0.01^c^	69.92 ± 0.02^a^	80.40 ± 0.02^d^	9.58 ± 0.02^c^	21.06 ± 0.10^c^	69.67 ± 0.05^b^
F_5_	82.18 ± 0.06^c^	11.85 ± 0.02^b^	22.77 ± 0.33^c^	68.75 ± 0.17^a^	83.67 ± 0.03^c^	13.59 ± 0.35^a^	25.61 ± 0.03^b^	66.72 ± 0.11^c^
F_6_	81.06 ± 0.04^c^	9.84 ± 0.23^c^	20.98 ± 0.12^c^	70.07 ± 0.10^a^	82.96 ± 0.04^c^	12.80 ± 0.10^b^	24.69 ± 0.01^b^	67.38 ± 0.02^c^

*Note*: Data are reported as average ± standard deviation. Values with different lowercase letters, according to Tukey's test, are significantly different in each column (*p* < 0.05).

The structure and constituent color of the formulation are effective factors that influence the crumb color of bread (Bolarinwa et al., 2019). Incorporation of goji berry powder was found to decrease the L* and b* indices and increase the a* index significantly (*p* < 0.05). The control sample with L* and WI values equal to 90.75 ± 0.02 and 70.74 ± 0.12, respectively, was the brightest sample. Composite breads containing goji berry powder are darker in color compared to control wheat bread, which is attributed to the beta‐carotene pigments of fruit. Among composite breads, those with 20% ww^−1^ of goji berry powder (F_4_) had the lowest L*, a*, and b* with values equal to 80.40 ± 0.02, 9.58 ± 0.02, and 21.06 ± 0.02, respectively. As can be seen from the specific volume data, the specific volume of bread samples increased to a level of 20% ww^−1^ (F_4_). This increase enhanced air bubbles and light scattering and ultimately led to a decrease in L* and an increase in opacity (Mohammadi et al., 2021). Results also indicated that with an increase in the level of goji berry powder incorporation (F_5_ and F_6_), the L* index has decreased despite the decrease in specific volume. It seems that at a higher incorporation level of goji berry, the color is more determined by formulation constituents than structure, which is also confirmed by the a* parameter. The WI changes in bread crumbs indicate a decrease in whiteness due to the increase in incorporation of goji berry powder, which is claimed to be induced by the dominance of fruit color, as demonstrated by Koca and Anil (2007).

### 3.6 Sensory analysis

**FIGURE 4:**
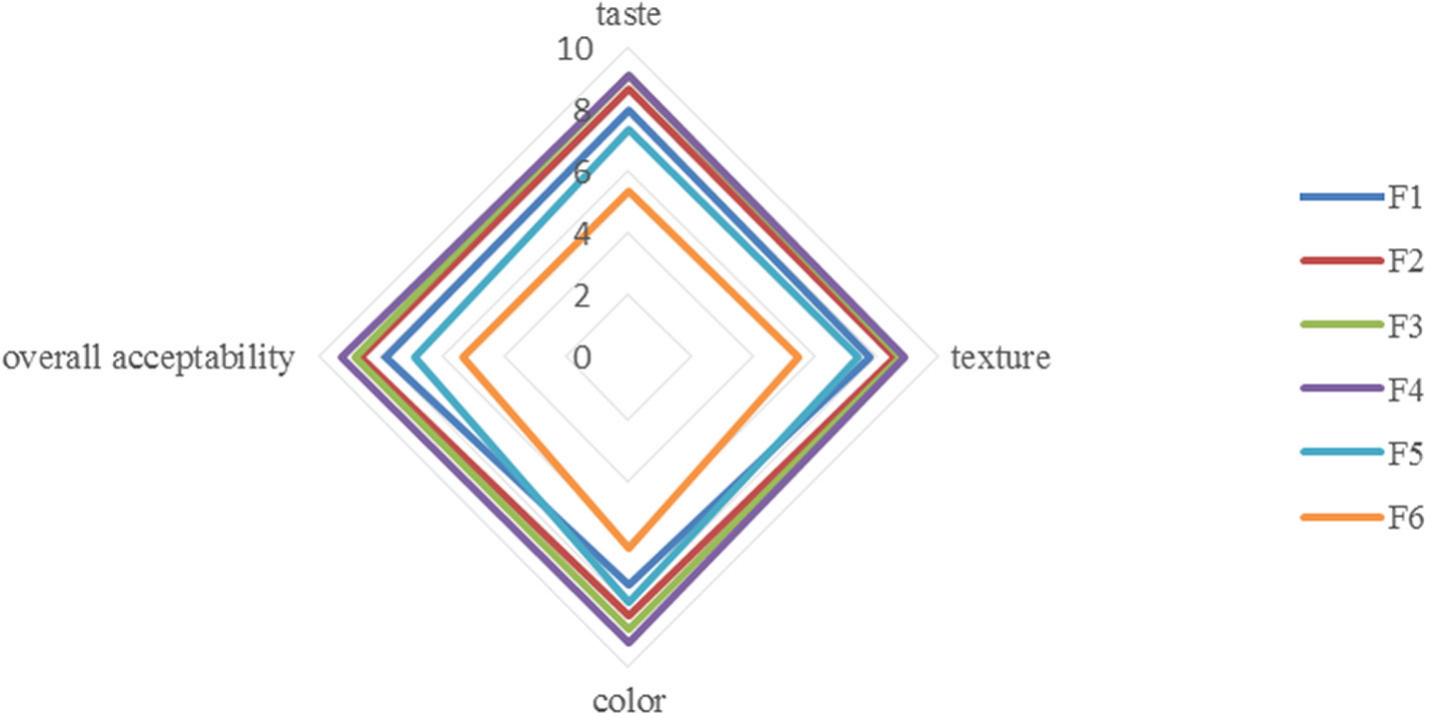
Radar plot for sensory evaluation of goji berry‐enriched wheat breads. Data are reported as average ± standard deviation.

A sensory assessment of composite bread prepared with goji berry powder is shown in Figure 4. According to the results obtained, at formulations containing goji berries higher than 20% ww^−1^, a decrease was observed in sensory acceptance by the consumer. In other words, at higher goji berry powder incorporation levels, increased darkness, decreased specific volume, and increased hardness, lead to a reduction in sensory acceptance. Among the samples containing goji berry powder, sample F_4_ was more preferred by consumers, with values equal to 8.92 ± 0.01 and 9.24 ± 0.03 for texture and overall acceptability, respectively, which has a significantly higher specific volume too. However, further incorporation of goji berry powder increases the amount of fiber in the formulation, which leads to the creation of a resinous structure with reduced specific volume and increased hardness, which reduces consumer satisfaction. The results showed that the overall acceptability had a good correlation with the specific volume.

The preferred perception for taste criteria is obtained by the F_3_ sample with no significant difference from the F_4_ sample (*p* ≥ 0.05). Due to the sour–sweet taste of goji berry fruit, adding it to wheat bread provides a pleasurable taste for consumers. Also, the sample with the highest amount of goji berry powder (F_6_) had an unpleasant taste that was not acceptable to consumers. Due to the desirable texture characteristics, taste, and color, the sample containing 20% ww^−1^ of goji berry powder (F_4_) had the most acceptable sample in terms of sensory parameters among consumers. However, higher inclusion levels had adversely changed the color, flavor, texture, and consequently the overall acceptability. A drop in bakery product sensory quality at a high substitute level by the addition of different fruit‐fibers, namely goji berry by‐products, mango peel, jack fruit, white grape pomace, and apple fiber, was also observed in previous studies (Bora et al., 2019; Feili et al., 2016; Mildner‐Szkudlarz et al., 2013; Pathak et al., 2016; Purić et al., 2020).

## 4 CONCLUSION

Goji berry, as a super fruit with proven nutritional and health effects, is potentially able to be incorporated in formulations of wheat bread. The current study suggests that goji berry incorporation at 20% ww^−1^ provides a gel‐like structure able to retain gases produced during the baking process, which is supposed to be induced by the fruit fiber. However, its incorporation at higher levels can noticeably deteriorate the sensory perception and textural characteristics of wheat bread, induced by the gluten dilution impacts. Increasing public awareness about the direct impact of diet on health and the approved health benefits of goji berries, as a native fruit of Asian countries, necessitates new product development.

## AUTHOR CONTRIBUTIONS

**Seyed Saba Hashemi:** Conceptualization (equal); investigation (equal); methodology (equal); resources (equal); validation (equal); writing – original draft (equal); writing – review and editing (equal). **Neda Mollakhalili‐Meybodi:** Data curation (equal); investigation (equal); project administration (equal); resources (equal); software (equal); validation (equal); writing – review and editing (equal). **Fateme Akrami Mohajeri:** Conceptualization (equal); funding acquisition (equal); investigation (equal); methodology (equal); resources (equal); validation (equal); visualization (equal); writing – review and editing (equal). **Hossein Fallahzadeh:** Conceptualization (equal); formal analysis (equal); methodology (equal); software (equal); supervision (equal); validation (equal); visualization (equal); writing – review and editing (equal). **Elham Khalili Sadrabad:** Conceptualization (equal); data curation (equal); investigation (equal); methodology (equal); project administration (equal); resources (equal); supervision (equal); validation (equal); writing – original draft (equal); writing – review and editing (equal).

## CONFLICT OF INTEREST STATEMENT

All authors declare no conflict of interest.

## Data Availability

The data that support the findings of this study are openly available by request from authors.

## References

[fsn34056-bib-0001] Ahmed, J. , Almusallam, A. S. , Al‐Salman, F. , AbdulRahman, M. H. , & Al‐Salem, E. (2013). Rheological properties of water insoluble date fiber incorporated wheat flour dough. LWT ‐ Food Science and Technology, 51(2), 409–416.

[fsn34056-bib-0003] American Association of Cereal Chemists. Approved Methods Committee . (2000). Approved methods of the American association of cereal chemists. AACC.

[fsn34056-bib-0004] Amin, M. N. , Hasan, M. N. , Zakariya, P. S. A. , Pralebda, S. A. , Pramono, H. , Saputra, E. , Subekti, S. , & Alamsjah, M. A. (2019). Texture profile of the bread produced from composite flour *Bruguiera gymnorrhiza* flour (BGF) and wheat flour. IOP Conference Series: Earth and Environmental Science, 236, 012109. 10.1088/1755-1315/236/1/012109

[fsn34056-bib-0005] Balabanova, T. , Ivanova, M. , Ivanov, I. , Dimitrova, M. , Dushkova, M. , & Vlaseva, R. (2018). Lactic acid beverage fortified with goji berry. Bulgarian Journal of Agricultural Science, 24(5), 885–890.

[fsn34056-bib-0006] Bolarinwa, I. F. , Aruna, T. E. , & Raji, A. O. (2019). Nutritive value and acceptability of bread fortified with moringa seed powder. Journal of the Saudi Society of Agricultural Sciences, 18(2), 195–200.

[fsn34056-bib-0007] Bora, P. , Ragaee, S. , & Abdel‐Aal, E.‐S. M. (2019). Effect of incorporation of goji berry by‐product on biochemical, physical and sensory properties of selected bakery products. LWT, 112, 108225.

[fsn34056-bib-0008] Codină, G. G. , Zaharia, D. , & Stroe, S.‐G. (2018). Influence of calcium ions addition from gluconate and lactate salts on refined wheat flour dough rheological properties. CyTA ‐ Journal of Food, 16(1), 884–891. 10.1080/19476337.2018.1498129

[fsn34056-bib-0009] Eliášová, M. , Kotíková, Z. , Lachman, J. , Orsák, M. , & Martinek, P. (2020). Influence of baking on anthocyanin content in coloured‐grain wheat bread. Plant, Soil and Environment, 66(8), 381–386.

[fsn34056-bib-0010] Encina‐Zelada, C. R. , Cadavez, V. , Monteiro, F. , Teixeira, J. A. , & Gonzales‐Barron, U. (2018). Combined effect of xanthan gum and water content on physicochemical and textural properties of gluten‐free batter and bread. Food Research International, 111, 544–555. 30007717 10.1016/j.foodres.2018.05.070

[fsn34056-bib-0011] Endes, Z. , Uslu, N. , Özcan, M. M. , & Er, F. (2015). Physico‐chemical properties, fatty acid composition and mineral contents of goji berry (*Lycium barbarum* L.) fruit. Journal of Agroalimentary Processes and Technologies, 21, 36–40.

[fsn34056-bib-0012] Feili, R. , Zzaman, W. , Nadiah, W. , Abdullah, W. , & Yang, T. A. (2013). Physical and sensory analysis of high fiber bread incorporated with jackfruit rind flour. Food Science and Technology, 1(2), 30–36.

[fsn34056-bib-0013] Gamel, T. H. , Abdel‐Aal, E.‐S. M. , & Tosh, S. M. (2015). Effect of yeast‐fermented and sour‐dough making processes on physicochemical characteristics of β‐glucan in whole wheat/oat bread. LWT ‐ Food Science and Technology, 60(1), 78–85.

[fsn34056-bib-0014] Guadarrama‐Lezama, A. Y. , Carrillo‐Navas, H. , Pérez‐Alonso, C. , Vernon‐Carter, E. J. , & Alvarez‐Ramirez, J. (2016). Thermal and rheological properties of sponge cake batters and texture and microstructural characteristics of sponge cake made with native corn starch in partial or total replacement of wheat flour. LWT, 70, 46–54.

[fsn34056-bib-0015] Guijarro‐fuertes, M. , Andrade‐cuvi, M. J. , Bravo‐vásquez, J. , Ramos‐guerrero, L. , & Vernaza, M. G. (2019). Andean blueberry (*Vaccinium floribundum*) bread: Physicochemical properties and bioaccessibility of antioxidants. Food Science and Technology, 39, 56–62.

[fsn34056-bib-0016] Hameed, W. , Gawlik‐dziki, U. , Alicja, S. , & Renata, R. (2021). The fruits of sumac (*Rhus coriaria* L.) as a functional additive and salt replacement to wheat bread. LWT, 136, 110346. 10.1016/j.lwt.2020.110346

[fsn34056-bib-0800] Iuga, M. , Boestean, O. , Ghendov‐Mosanu, A. , & Mironeasa, S. (2020). Impact of dairy ingredients on wheat flour dough rheology and bread properties. Foods, 9(6), 828. 32599829 10.3390/foods9060828 PMC7353663

[fsn34056-bib-0017] Iuga, M. , & Mironeasa, S. (2020). Potential of grape byproducts as functional ingredients in baked goods and pasta. Comprehensive Reviews in Food Science and Food Safety, 19(5), 2473–2505. 33336974 10.1111/1541-4337.12597

[fsn34056-bib-0018] Jagelaviciute, J. , & Cizeikiene, D. (2021). The influence of non‐traditional sourdough made with quinoa, hemp and chia flour on the characteristics of gluten‐free maize/rice bread. LWT, 137, 110457.

[fsn34056-bib-0019] Kim, H. W. , Lee, I. J. , Park, S. M. , Lee, J. H. , Nguyen, M.‐H. , & Park, H. J. (2019). Effect of hydrocolloid addition on dimensional stability in post‐processing of 3D printable cookie dough. LWT, 101, 69–75.

[fsn34056-bib-0020] Koca, A. F. , & Anil, M. (2007). Effect of flaxseed and wheat flour blends on dough Rheology and bread quality. Journal of the Science of Food and Agriculture, 87(6), 1172–1175.

[fsn34056-bib-0021] Koçyiğit, E. , & Şanlier, N. (2017). A review of composition and health effects of *Lycium barbarum* . International Journal of Chinese Medicine, 1, 1–9.

[fsn34056-bib-0022] Kohajdová, Z. , Karovičová, J. , Magala, M. , & Kuchtová, V. (2014). Effect of apple pomace powder addition on farinographic properties of wheat dough and biscuits quality. Chemical Papers, 68(8), 1059–1065. 10.2478/s11696-014-0567-1

[fsn34056-bib-0023] Korus, J. , Witczak, M. , Ziobro, R. , & Juszczak, L. (2017). Hemp (cannabis sativa Subsp. Sativa) flour and protein preparation as natural nutrients and structure forming agents in starch based gluten‐free bread. LWT, 84, 143–150.

[fsn34056-bib-0024] Kou, X. , Luo, D. , Zhang, K. , Wei, X. , Li, X. , Baocheng, X. , Li, P. , Han, S. , & Liu, J. (2019). Textural and staling characteristics of steamed bread prepared from soft flour added with inulin. Food Chemistry, 301, 125272. 31377629 10.1016/j.foodchem.2019.125272

[fsn34056-bib-0025] Kowalczewski, P. Ł. , Walkowiak, K. , Masewicz, Ł. , Duda, A. , Poliszko, N. , Różańska, M. B. , Jeżowski, P. , Tomkowiak, A. , Mildner‐Szkudlarz, S. , & Baranowska, H. M. (2019). Wheat bread enriched with raspberry and strawberry oilcakes: Effects on proximate composition, texture and water properties. European Food Research and Technology, 245, 2591–2600.

[fsn34056-bib-0026] Lauková, M. , Kohajdová, Z. , Karovičová, J. , Kuchtová, V. , Minarovičová, L. , & Tomášiková, L. (2017). Effects of cellulose fiber with different fiber length on rheological properties of wheat dough and quality of baked rolls. Food Science and Technology International, 23(6), 490–499. 28399637 10.1177/1082013217704122

[fsn34056-bib-0027] Liu, Y. , Leng, Y. , Xiao, S. , Zhang, Y. , Ding, W. , Ding, B. , Yan, W. , Wang, X. , & Yang, F. (2022). Effect of inulin with different degrees of polymerization on dough Rheology, gelatinization, texture and protein composition properties of extruded flour products. LWT, 159, 113225.

[fsn34056-bib-0028] Loizzo, M. R. , Mincione, A. , Tundis, R. , & Sicari, V. (2021). Enrichment of bread with *Lycium barbarum* (goji) puree. Biology and Life Sciences Forum, 6, 1.

[fsn34056-bib-0029] Martínez, M. M. , & Gómez, M. (2017). Rheological and microstructural evolution of the Most common gluten‐free flours and starches during bread fermentation and baking. Journal of Food Engineering, 197, 78–86.

[fsn34056-bib-0030] McCleary, B. V. , Sloane, N. , Draga, A. , & Lazewska, I. (2013). Measurement of total dietary fiber using AOAC Method 2009.01 (AACC International Approved Method 32‐45.01): Evaluation and updates. Cereal Chemistry, 90, 396–414.

[fsn34056-bib-0031] Meybodi, N. M. , Mortazavian, A. M. , Mirmoghtadaie, L. , Hosseini, S. M. , Yasini, S. A. , Azizi, M. H. , & Nodoushan, S. M. (2019). Effects of microbial transglutaminase and fermentation type on improvement of lysine availability in wheat bread: A response surface methodology. Applied Food Biotechnology, 6(3), 151–164.

[fsn34056-bib-0032] Mildner‐Szkudlarz, S. , Bajerska, J. , Górnaś, P. , Segliņa, D. , Pilarska, A. , & Jesionowski, T. (2016). Physical and bioactive properties of muffins enriched with raspberry and cranberry pomace powder: A promising application of fruit by‐products rich in biocompounds. Plant Foods for Human Nutrition, 71(2), 165–173. 27037934 10.1007/s11130-016-0539-4 PMC4891392

[fsn34056-bib-0700] Mildner‐Szkudlarz, S. , Bajerska, J. , Zawirska‐Wojtasiak, R. , & Górecka, D. (2013). White grape pomace as a source of dietary fibre and polyphenols and its effect on physical and nutraceutical characteristics of wheat biscuits. Journal of the Science of Food and Agriculture, 93(2), 389–395. 22806270 10.1002/jsfa.5774

[fsn34056-bib-0034] Mohammadi, F. , Ehrampoush, M. H. , Shamsi, F. , Ardakani, S. A. Y. , & Mollakhalili‐Meybodi, N. (2021). Inulin enriched wheat bread: Interaction of polymerization degree and fermentation type. Journal of Food Measurement and Characterization, 15(6), 5408–5417.

[fsn34056-bib-0035] Pathak, D. , Majumdar, J. , & Raychaudhuri, U. (2016). Characterization of physicochemical properties in whole wheat bread after incorporation of ripe mango peel. Journal of Food Measurement and Characterization, 10, 554–561. 10.1007/s11694-016-9335-y

[fsn34056-bib-0036] Purić, M. , Rabrenović, B. , Rac, V. , Pezo, L. , Tomašević, I. , & Demin, M. (2020). Application of defatted apple seed cakes as a by‐product for the enrichment of wheat bread. LWT, 130, 109391. 10.1016/j.lwt.2020.109391

[fsn34056-bib-0037] Ragaee, S. , Guzar, I. , Dhull, N. , & Seetharaman, K. (2011). Effects of fiber addition on antioxidant capacity and nutritional quality of wheat bread. LWT ‐ Food Science and Technology, 44(10), 2147–2153.

[fsn34056-bib-0038] Shiri, A. , Ehrampoush, M. H. , Ardakani, S. A. Y. , Shamsi, F. , & Mollakhalili‐Meybodi, N. (2021). Technological characteristics of inulin enriched gluten‐free bread: Effect of acorn flour replacement and fermentation type. Food Science & Nutrition, 9(11), 6139–6151. 34760245 10.1002/fsn3.2567 PMC8565209

[fsn34056-bib-0039] Shittu, T. A. , Egwunyenga, R. I. , Sanni, L. O. , & Abayomi, L. (2014). Bread from composite plantain‐wheat flour: I. Effect of plantain fruit maturity and flour mixture on dough rheology and fresh loaf qualities. Journal of Food Processing and Preservation, 38, 1821–1829. 10.1111/jfpp.12153

[fsn34056-bib-0040] Skenderidis, P. , Lampakis, D. , Giavasis, I. , Leontopoulos, S. , Petrotos, K. , Hadjichristodoulou, C. , & Tsakalof, A. (2019). Chemical properties, fatty‐acid composition, and antioxidant activity of goji berry (*Lycium barbarum* L. and *Lycium chinense* Mill.) fruits. Antioxidants, 8, 60. 30857360 10.3390/antiox8030060 PMC6466590

[fsn34056-bib-0041] Struck, S. , Straube, D. , Zahn, S. , & Rohm, H. (2018). Interaction of wheat macromolecules and berry pomace in model dough: Rheology and microstructure. Journal of Food Engineering, 223, 109–115. 10.1016/j.jfoodeng.2017.12.011

[fsn34056-bib-0042] Tamba‐Berehoiu, R.‐M. , Turtoi, M. O. , Vișan, L. V. , & Popa, C. (2017). Physico‐chemical, rheological and technological characterization of some mixtures of wheat, oat, barley and millet flours. The Annals of the University Dunarea De Jos of Galati. Fascicle VI ‐ Food Technology, 41(2), 102–114.

[fsn34056-bib-0043] Valková, V. , Ďúranová, H. , Štefániková, J. , Miškeje, M. , Tokár, M. , Gabríny, L. , Kowalczewski, P. Ł. , & Kačániová, M. (2020). Wheat bread with grape seeds micropowder: Impact on dough rheology and bread properties. Applied Rheology, 30(1), 138–150.

[fsn34056-bib-0044] Wenli, S. , Shahrajabian, M. H. , & Qi, C. (2019). Therapeutic roles of goji berry and ginseng in traditional Chinese. Journal of Nutrition and Food Security, 4, 293–305.

[fsn34056-bib-0045] Witczak, T. , Juszczak, L. , Ziobro, R. , & Korus, J. (2017). Rheology of gluten‐free dough and physical characteristics of bread with potato protein. Journal of Food Process Engineering, 40(3), e12491.

[fsn34056-bib-0046] Xu, J. , Li, Y. , Zhao, Y. , Wang, D. , & Wang, W. (2021). Influence of antioxidant dietary fiber on dough properties and bread qualities: A review. Journal of Functional Foods, 80, 104434.

[fsn34056-bib-0047] Zhu, F. , Sakulnak, R. , & Wang, S. (2016). Effect of black tea on antioxidant, textural, and sensory properties of Chinese steamed bread. Food Chemistry, 194, 1217–1223. 26471674 10.1016/j.foodchem.2015.08.110

